# Address of Greeting

**Published:** 1867-06

**Authors:** 


ADDRESS OF GREETING.

*Mr. President and Gentlemen of the Association:*

It is my pleasant duty to greet you on this occasion, and to give you a cordial welcome to this city. I welcome you also on behalf of the profession, and of the citizens at large.

Seventeen years ago this Association honored us with a meeting. Now, as then, we are happy in having the pleasure of greeting representatives from all parts of our beloved country.

It is, then, sir, with feelings of no ordinary pleasure, that I welcome this Association—American by name, national and catholic in spirit—once more to the hospitalities of our city. Its history is bright with the names and labors of the great and good in all parts of our country.

It has harmonized the profession, elevated its tone, stimulated a desire for a higher standard of medical education, and above all, has drawn a line as of fire between the scientific physician and the empyric, by adopting the Code of Ethics.

Its power for good is hardly to be estimated. Its yearly transactions have received high commendation. No man in the profession can be indifferent to it. Much remains to be done to make its labors still more valuable.

Without any power from the State or the National Government, to execute its mandates, it must for the future, as in the past, rely on the union, enthusiasm, and scientific labors of its members.

Having the highest confidence in the capacity of the Association for usefulness, and trusting that its labors may be still more conducive to the advancement of the science and improvement of the art, I bid you God speed in your efforts, and again most heartily welcome you to our city and our homes, as distinguished and honored guests.

